# Death of the Emperor Napoleon

**Published:** 1873-03

**Authors:** 


### DEATH OF THE EMPEROR NAPOLEON.

The ex-Emperor was in his sixty-fifth year, and had long suffered from phosphatic calculus with local catarrhal symptoms. The operation of lithotrity was performed by Sir Henry Thompson, the eminent surgeon. An account of his last days appears in the various English medical periodicals, from which we extract as follows:

A first operation was performed January 2d, and on Monday 6th, a second. The results were satisfactory. In the words of the bulletin of January 6th, issued at 2 P. M., two hours after the operation, "The difficulties were greater than usual, but the results obtained were considerable. There is much suffering, together with some degree of constitutional disturbance, but the general strength remains good." On the morning of the 7th, a comparatively favorable announcement was made, the night having been fairly tranquil; but the symptoms of irritation again recurred, and the afternoon bulletin indicated the persistence of unfavorable conditions. The morning of Wednesday opened unsatisfactorily, the night having been passed in much pain. In the words of the bulletin, "The local symptoms are more severe, but the general state remains as yesterday." Here may be traced the effect of an obstructing mass of calculus, which was so lodged as to cause local distress and much reflex irritation. Under these circumstances, measures were taken in preparation for a further sitting, intended to remove the obstruction and to give relief. The night of Wednesday passed very quietly, and Mr. Cooper Forster and Mr. Clover, who had been summoned to Chiselhurst by Sir Henry Thompson, remained in view of an operation on the next day.

At eleven o'clock on Wednesday night, at 2 A.M. and 6 A.M. on Thursday morning, the Emperor was found, when visited by his medical attendants, to be sleeping naturally and soundly, and to be altogether better than on the previous days. At a little before ten he was still in a state of comparative ease, and every thing promised well. The proposed sitting was accordingly arranged for noon. But at 10:25 he was found to be suddenly sinking; he became unconscious; the pulse, which had previously been at 84, rapidly fell; and the heart failed in its action,

The signs of sudden prostration and rapidly approaching dissolution appeared. Nor were any means which could be employed in any degree successful in averting a suddenly fatal result. It was evident that death was occurring, either from a rapid failure of action of the heart or from arrest of the circulation by a blood-clot. Death followed within twenty minutes from the first seizure, the Emperor having been unconscious throughout, and of course also insensible to pain and spared from the pangs of death. It was hard, even for those who are taught by professional discipline to preserve their whole powers intact and alert during such emergencies, to realize the sad truth. For the agonized wife, to whom such bereavement seemed physically as well as morally incredible and overwhelming, its suddenness and tranquility may well have seemed to add an additional pang to her moral suffering. "*C'est impossible*" has been before the cry of more than one widowed wife whom the sudden stroke of death has thus robbed of one who was nearest to her.

Such a termination to this history painfully illustrates the uncertainties which beset and the perils which underlie our art. It has nothing in common with lithotrity as such, but is a fatal incident, such as is recorded from time to time in conditions of apparent health, and also after any kind of operation. The mortality of lithotrity is very small; and this is not among the fatal accidents which belong to it.

